# The Role of Catecholamines in Pathophysiological Liver Processes

**DOI:** 10.3390/cells11061021

**Published:** 2022-03-17

**Authors:** Elise Lelou, Anne Corlu, Nicolas Nesseler, Claudine Rauch, Yannick Mallédant, Philippe Seguin, Caroline Aninat

**Affiliations:** 1 INSERM, Université Rennes, INRAE, Institut NuMeCan, Nutrition, Metabolisms and Cancer, F-35000 Rennes, France; elise.lelou@univ-rennes1.fr (E.L.); anne.corlu@univ-rennes1.fr (A.C.); nicolas.nesseler@chu-rennes.fr (N.N.); claudine.rauch@univ-rennes1.fr (C.R.); yannick.malledant@chu-rennes.fr (Y.M.); philippe.seguin@chu-rennes.fr (P.S.); 2 CHU Rennes, Department of Anesthesia and Critical Care, F-35000 Rennes, France

**Keywords:** hepatic cells, epinephrine, norepinephrine, dopamine, sympathetic innervation

## Abstract

Over the last few years, the number of research publications about the role of catecholamines (epinephrine, norepinephrine, and dopamine) in the development of liver diseases such as liver fibrosis, fatty liver diseases, or liver cancers is constantly increasing. However, the mechanisms involved in these effects are not well understood. In this review, we first recapitulate the way the liver is in contact with catecholamines and consider liver implications in their metabolism. A focus on the expression of the adrenergic and dopaminergic receptors by the liver cells is also discussed. Involvement of catecholamines in physiological (glucose metabolism, lipids metabolism, and liver regeneration) and pathophysiological (impact on drug-metabolizing enzymes expression, liver dysfunction during sepsis, fibrosis development, or liver fatty diseases and liver cancers) processes are then discussed. This review highlights the importance of understanding the mechanisms through which catecholamines influence liver functions in order to draw benefit from the adrenergic and dopaminergic antagonists currently marketed. Indeed, as these molecules are well-known drugs, their use as therapies or adjuvant treatments in several liver diseases could be facilitated.

## 1. Introduction

Catecholamines (epinephrine, norepinephrine, dopamine) are well-known neurotransmitters implicated in several central functions such as memory process, emotions, and cognition. In the periphery, they are considered as neurohormones and are mainly involved in the fight-or-flight response that allows the organism to react rapidly to a stressful situation. In the liver, the initial research focused on their physiological role, such as the regulation of carbohydrates by epinephrine or liver regeneration, whereas more recent works have dealt with their involvement in pathophysiological processes (such as their involvement in the apparition of non-alcoholic fatty liver diseases or in the progression of liver cancers). In this review, we recapitulate the physiological aspects of catecholamines and then discuss their implication in diseases.

## 2. How the Liver Is in Contact with Catecholamines?

### 2.1. Liver Sympathetic Innervation

Postganglionic sympathetic innervation from the celiac and superior mesenteric ganglia enters the liver by the hilus and forms two nervous plexuses, the anterior and the posterior plexus, following the branching of the hepatic artery and the portal vein, respectively. This innervation has been recently illustrated in elegant works using the immunolabeling-enabled 3D imaging of solvent-cleared organs (iDISCO) technique [1,2]. In humans, sympathetic fibers have been detected in the entire intralobular region of the liver parenchyma, and terminations of these nerves are observed close to the blood vessels as well as in the space of Disse, allowing direct contact with hepatocytes, stellate cells, and sinusoidal endothelial cells [3,4,5,6]. However, the distribution is different in rodent livers, where the nerve fibers’ localization is limited to the portal tracts of interlobular regions [5,7]. In humans, sympathetic innervations are also present in the bile ducts [1,2]. The concentration of norepinephrine in human livers has been estimated to be around 2.18 nmol per g of wet tissue [8]. The main source of norepinephrine in this organ appears to be the sympathetic nerve fibers since, after liver transplantation, where denervation is done, its concentration dips to 0.019 nmol per g of wet tissue [8]. These results are less contrasted with epinephrine as the concentration in the liver drops from 0.04 to 0.01 nmol per g of wet tissue before and after transplantation, respectively. Of note, although dopaminergic nerves have not been detected in the hepatic parenchyma [9], hepatic cells can be in contact with dopamine, given that sympathetic nerve fibers are also able to release dopamine in addition to norepinephrine.

The release of catecholamines from sympathetic endings is under the control of specific zones in the hypothalamus. Two mechanisms can explain the stimulation of these zones:

I. Neurons present in these locations are sensitive to several peripheral hormones such as leptin, insulin, or glucagon-like peptide 1. Glucose-sensing neurons are also present in these zones. Once activated, these neurons can stimulate sympathetic nerve fibers ending in the liver, leading to a local release of norepinephrine [10,11];
- II. Afferent vagal and spinal nerves leave the liver to specific regions of the hypothalamus. These afferent nerves can be activated by several stimuli such as glucose, metabolites, pH, and cytokines. In response to these stimulations, neurons present in the hypothalamus activate efferent sympathetic fibers, leading to the release of norepinephrine in the periphery [12].

### 2.2. Adrenal Gland and Mesenteric Organs Are the Main Sources of Blood Delivering Catecholamines to the Liver

The adrenal medulla releases epinephrine in the circulation in response to several stressors (e.g., hypoglycemia, fear, hypotension, hypoxia). Once in the bloodstream, epinephrine can reach the liver. Concerning norepinephrine, a very tiny fraction is released into the circulation by the sympathetic nerves that innervate organs and blood vessels [13]. Among these organs, mesenteric organs are an important source of norepinephrine for the liver as secretion occurs directly in the portal tract [14]. In addition to norepinephrine, the gastrointestinal tract, spleen, and pancreas are also significant sources of dopamine due to its synthesis by noradrenergic nerves, tyrosine hydroxylase-containing cells, and non-neuronal cells [15].

### 2.3. Hepatic Stellate Cells: A Source of Catecholamines?

Some studies have described catecholamines synthesis in activated stellate cells. Firstly, murine stellate cells express tyrosine hydroxylase and dopamine-*β*-hydroxylase and both norepinephrine and dopamine have been detected in the culture medium of these cells [16]. Secondly, the secretion of norepinephrine and, to a lower level, epinephrine by human stellate cells has also been observed [17]. In these cultures, endogenous synthesis of norepinephrine has an autocrine action by allowing cell proliferation and by having an anti-apoptotic effect [16,17]. These in vitro results suggest that activation of hepatic stellate cells leads to the synthesis of catecholamines, which would not be observed in quiescent cells. On the other hand, single-cell RNA sequencing analysis of 246 hepatic stellate cells has recently revealed the presence of two hepatic stellate cell populations in the human liver. One of these populations is located in the portal and central vein area. The second is specifically present in the perisinusoidal space and is characterized by a specific signature with a high expression of dopamine *β*-hydroxylase [18]. This result raises the possibility that not all hepatic stellate cells produce catecholamines.

In addition, at least two publications have raised the possibility of an epinephrine biosynthesis from norepinephrine by the liver related to nonspecific N-methyltransferases [19,20]. This possibility of catecholamines synthesis by stellate cells is quite interesting and deserves to be supported by other works in order to fully understand its relevance in pathophysiological process.

## 3. Catecholamines Metabolism in the Liver

As previously mentioned, the liver is in contact with catecholamines and seems able to synthesize them. However, the liver is also known to play an important role in their metabolism. Thus, epinephrine and norepinephrine are carried in hepatocytes by various transporters such as organic cation transporter (OCT) 1, 2, and 3 and uptake2 and P-glycoprotein [21,22]. They are then metabolized directly by the monoamine oxidases (MAOs) to give a reactive intermediate product, 3,4-dihydroxyphenylglycoaldehyde (DOPEGAL) [23]. This compound is bio-transformed in 3,4-dihydroxyphenylglycol (DHPG) by the aldehyde reductases. DHPG, a substrate of the catechol-*O*-methyltransferase (COMT), gives 4-hydroxy-3-methoxyphenylglycol (MHPG), which, in turn, is metabolized in vanillylmandelic acid (VMA) by the action of the alcohol dehydrogenase and the aldehyde dehydrogenase [24]. However, in hepatocytes, epinephrine and norepinephrine are also directly metabolized by the COMT in metanephrine (MN) and normetanephrine (NMN), respectively [25,26]. MN and NMN are taken in charge by the MAO and the aldehyde reductases to form the MHPG, which is then metabolized in VMA, as previously described.

**Figure 1:**
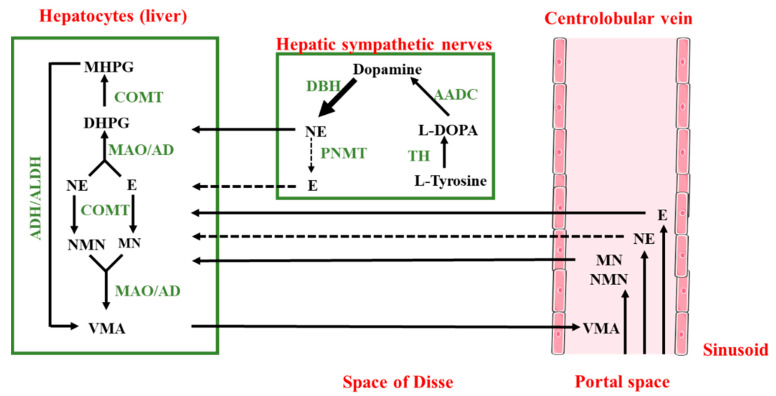
Metabolism of epinephrine (E) and norepinephrine (NE) in the liver. Adrenal glands, mesenteric organs, and liver sympathetic innervation are the main sources of E and NE in the liver. The last step of metabolism for metanephrine (MN) and normetanephrine (NMN) extracted from the circulation are also performed in the liver. Thus, E, NE, MN, and NMN are uptaken by hepatocytes and metabolized in VMA. VMA is then eliminated in the urine. AD: aldehyde reductase; ADH: alcohol dehydrogenase; ALDH: aldehyde dehydrogenase; MAO: monoamine oxidases; COMT: catechol-O-methyltransferase; TH: tyrosine hydroxylase; AADC: aromatic L-aminoacid decarboxylase; DBH: dopamine-*β*-hydroxylase; PNMT: phenylethanolamine N-methyltransferase; DHPG: 3,4-dihydroxyphenylglycol; MHPG: 4-hydroxy-3methoxyphenylglycol; VMA: vanillyl mandelic acid (Solid arrow: main pathway; dotted arrow: minor pathway).

Besides the role of the liver in their metabolism, the catecholamines are mainly metabolized on their sites of production. Because their metabolites are released in the blood circulation, NMN, MN, MHPG, and DHPG are produced in extra-neuronal tissues; sympathetic nerve fibers or adrenal glands can also be carried in hepatocytes. After their uptake by the liver, they are mainly transformed in VMA before being eliminated via the kidney in urines [14]. Accordingly, the main metabolite of catecholamines excreted in the urine is VMA, and more than 94% is produced within the liver [15]. Sulfated metabolites of DHPG, MHPG, and NMN are also found in the portal circulation [27,28]. They are formed by the sulfotransferase 1A3 (SULT1A3) expressed in the mesenteric organs [29,30], and some of them can be eliminated in the bile by the multidrug resistance-associated protein (MRP) 2 transporter [31]. The main pathways of this metabolism are summarized in Figure 1.

Dopamine is rapidly conjugated in mesenteric organs to dopamine-sulfate by SULT1A3, and this sulfoconjugate is found at high concentrations in plasma: more than 90% of dopamine is found in a sulfoconjugated form in the circulation [13,32,33]. The UDP-glucuronosyltransferase 1A10 (UGT1A10), expressed in epithelial cells of the gastrointestinal tract, is also able to form dopamine 3- and 4-glucuronates from dopamine [34]. Interestingly, in mouse liver, very low concentrations of dopamine metabolites are observed [35]. This could be due to the fact that dopamine is mainly used as a precursor of norepinephrine and, consequently, only poorly secreted by sympathetic termination.

Interestingly, an enterohepatic cycle has been identified for dopamine- and norepinephrine-conjugates in an experience using germ-free mice. Thus, once eliminated in the duodenum with the bile, catecholamine-conjugates could be deconjugated by bacteria, generating free catecholamines in the duodenal lumen. These free catecholamines, in addition to being reabsorbed, could have an impact on gut microorganisms [36].

## 4. Adrenergic and Dopaminergic Receptors Expression in the Liver

**Figure 2:**
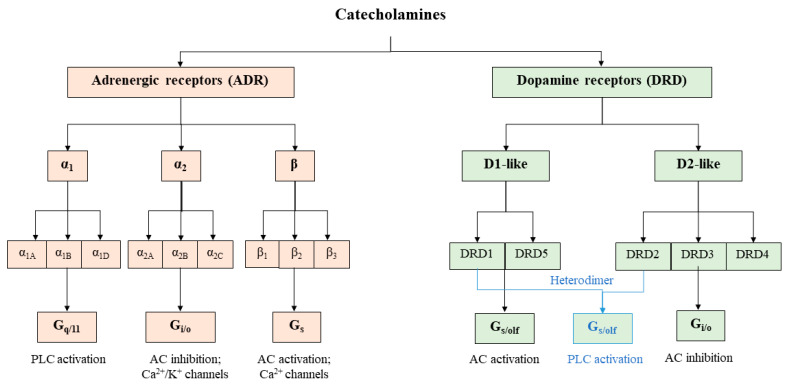
Catecholamines receptors. Catecholamines can activate adrenergic receptors (ADRs), composed of 9 receptors (*α*_1A_, *α*_1B_, *α*_1D_, *α*_2A_, *α*_2B_, *α*_2C_, *β*_1_, *β*_2,_ and *β*_3_), or dopamine receptors (DRDs), composed of 5 receptors (DRD1 to 5). Each receptor is coupled to G proteins alpha (q/11; i/o; s; s/olf) that can activate or inhibit effectors such as phospholipase C (PLC), adenylate cyclase (AC), or ion channels.

Catecholamines are ligands of G-protein-coupled receptors (GPCRs). Dopamine binds to dopamine receptors (DRDs), and both norepinephrine and epinephrine bind to adrenergic receptors (ADRs) (Figure 2). However, these catecholamines are not exclusive to their specific receptors as dopamine can activate ADRs and both norepinephrine and epinephrine can activate DRDs [37,38,39,40].

### 4.1. Dopamine Receptors

There are five subtypes of DRD, namely, *DRD1* to *DRD5*, encoded by five distinct genes. They are divided into two groups. The first one includes the D1-like receptors (DRD1 and DRD5) that are coupled to Gs*α* proteins. By stimulating adenylate cyclase (AC), they lead to an increase in the cyclic adenosine monophosphate (cAMP) intracellular level. The second group is composed of D2-like receptors (DRD2 to DRD4), which are coupled to Gi*α* proteins and allow the decrease of the cAMP level by inhibiting AC (Figure 2). DRDs are mainly expressed in dopaminergic neurons and, to a lesser extent, in the kidney and the heart. According to the public Human Protein Atlas (www.proteinatlas.org; accessed on 10 December 2021), DRDs do not seem to be expressed at mRNA and protein levels in hepatocytes, stellate cells, Kupffer cells, or sinusoidal endothelial cells. However, four human cholangiocarcinoma cell lines (Mz-ChA-1, HuCCT-1, SG231, and CCLP-1), as well as the non-malignant H69 cholangiocyte cell line, express all *DRD*s [41]. Moreover, DRD2 is expressed in rat cholangiocytes on the basolateral membrane [42]. The human HepG2 hepatoma cell line expresses the five receptors, whereas only the DRD1 and DRD5 are present in the human Hep3B hepatoma cell line [43,44]. To further characterize DRD expression in hepatocytes, we analyzed their expression by RT-qPCR in other hepatoma cell lines (HepaRG, HBG-BC2, HepG2, and HuH7) as well as in human hepatocytes in primary culture (Table 1 and Figure S1). We found a very low expression for all these receptors in all these cell lines (Table 1). However, *DRD4* and *DRD5* seem to have higher expression in HepG2 cells and in HepaRG-progenitors and -hepatocytes in our culture conditions. Thus, our data and others indicate that DRDs could be expressed in hepatocytes and cholangiocytes; however, the cues responsible for their expressions remain to be characterized.

### 4.2. Adrenergic Receptors

Nine ADRs encoded by different genes are identified: *ADRA1A, ADRA1B,* *ADRA1D, ADRA2A, ADRA2B, ADRA2C, ADRB1, ADRB2,* and *ADRB3*. The *α*_1_-ADR (*ADRA1A*, *ADRA1B*, and *ADRA1D*) are coupled to G_q/11_ proteins and are able to increase intracellular Ca^2+^ levels through the phospholipase C (PLC) pathway. The *α*_2_-ADR (*ADRA2A*, *ADRA2B*, and *ADRA2C*) are coupled to G_iα_ proteins and inhibit the AC, whereas the *β*-ADR (*ADRB1*, *ADRB2*, and *ADRB3*) are mainly coupled to G_s*α*_ proteins and stimulate the AC (Figure 2). These adrenoceptors are functional as homodimers or heterodimers. Heterodimerization happens between different classes of ADRs. For example, *α*_1_-ADR is able to heterodimerize with *β*_2_-ADR [45].

**Figure 3:**
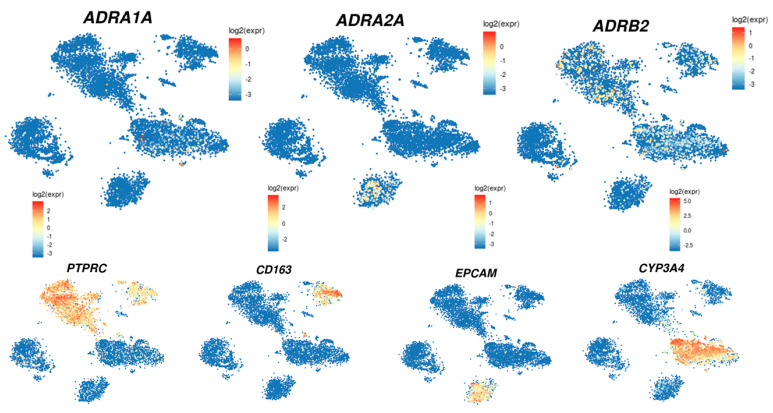
Analysis of *ADRA1A*, *ADRA2A,* and *ADRB2* expression using the single cell-RNA sequencing of normal human liver. Plot gene expression in the t-SNEP map of single-cell transcriptomes obtained from 9 normal human liver tissues was accessible on the open web interface (http://human-liver-cell-atlas.ie-freiburg.mpg.de/; accessed on 3 March 2022). *ADRA1A*, *ADRA2A,* and *ADRB2* expression were analyzed. PTPRC was used as a marker of leukocytes, CD163 as a marker of macrophage and Kupffer cell populations, EPCAM as a marker of the cholangiocyte population, and CYP3A4 as a marker of the hepatocyte population [46].

In the liver, those receptors are differentially expressed depending on the cell types, i.e., stellate cells, Kupffer cells, cholangiocytes, or hepatocytes (Table 2). The analysis of their expression using the single cell-RNA sequencing of the human liver (http://human-liver-cell-atlas.ie-freiburg.mpg.de/; accessed on 3 March 2022) [46] reveals that *ADRA1A*, *ADRA2A,* and *ADRB2* are the most expressed in normal liver. Interestingly, whereas *ADRA1A* and *ADRB2* seem mainly expressed in the hepatocytes, *ADRB2* is also expressed in leucocytes and, according to the cluster identified by Aizarani et al. (2019) [46], mainly in NK, NKT, and T-cell populations as well as in Kupffer cells. On the other hand, *ADRA2A* seems mainly expressed in the cholangiocyte population (Figure 3).

Interestingly, the density and expression of adrenoceptors can change through aging. The *β*_2_-ADR expression is high in fetal rat liver and rapidly decreases in postnatal days, whereas the opposite is observed for *α*_1_-ADR [47]. However, during aging, an increase in the expression of *β*-ADR is observed in rodents [47,48,49].

**Table 2:** Adrenoceptors expression in liver cells.

ADR Subtype	Hepatic Cells or Cell Lines	References	Methods Used
*α*_1A_	Human hepatic stellate cells	[50]	RT-PCR
	Activated Human hepatic stellate cells	[17]	RT-PCR; Western blot
	Human liver	[17]	RT-PCR,
		[51]	RNA-seq
	LO2 cells++	[50]	RT-PCR, Western blot
		[51]	
		[52]	
	HepG2 cells	[51]	RT-PCR, Western blot
		[52]	RT-PCR, Western blot
	HuH7 cells	[51]	RT-PCR, Western blot
	MHCC97 cells	[52]	RT-PCR, Western blot
	SK-Hep1, PLC/PRF/5, Snu423 cells	[51]	RT-PCR, Western blot
	FIH and PHH (high expression)	[53]	qPCR
	HepaRG cell (high expression)	[53]	qPCR
	Rat Kupffer Cells	[54]	Immunofluorescence
	Rat cholangiocytes	[55]	Immunohistochemistry
*α*_1B_	Rat liver	[56]	Northern blot
	Human liver	[50]	RT-PCR
		[51]	RNA-seq
	FIH and PHH (low expression)	[53]	qPCR
	LO2, HepG2 and MCC98H cells	[52]	RT-PCR
	Mice hepatic stellate cells	[16]	RT-PCR
	Rat Kupffer Cells	[54]	Immunofluorescence
	Rat cholangiocytes	[55]	Immunohistochemistry
*α*_1D_	Mice hepatic stellate cells	[16]	RT-PCR
	Rat Kupffer Cells	[54]	Immunofluorescence
	Rat cholangiocytes	[55]	Immunohistochemistry
*α*_2A_	Human liver	[50]	RT-PCR
		[51]	RNA-seq
	Rat Kupffer Cells	[54]	Immunofluorescence
*α*_2B_	Human hepatic stellate cells	[50]	RT-PCR
	Human liver	[50]	RT-PCR
		[51]	RNA-seq
	FIH (low expression)	[53]	qPCR
	Rat Kupffer Cells	[54]	Immunofluorescence
	Rat liver	[56]	Northern blot
*α*_2C_	FIH and PHH (low expression)	[53]	qPCR
	Rat Kupffer Cells	[54]	Immunofluorescence
*β*_1_	Human liver	[50]	RT-PCR
		[51]	RNA-seq
	Activated Human hepatic stellate cells	[17]	RT-PCR; Western blot
	FIH and HepaRG cells (low expression)	[53]	qPCR
	Mice hepatic stellate cells	[16]	RT-PCR
	Rat Kupffer Cells	[54]	Immunofluorescence
*β*_2_	Rat liver	[56]	Northern blot
		[50]	RT-PCR
	Human liver	[57]	Western blot
		[51]	RNA-seq
		[58]	Immunohistochemistry
		[50]	RT-PCR
	Human hepatic stellate cells	[50]	RT-PCR
	Activated Human hepatic stellate cells	[17]	RT-PCR; Western Blot
		[52]	Western blot; RT-PCR
	LO2 cells +	[59]	Western blot; RT-PCR
	HepG2 cells ++	[52]	Western blot; RT- PCR
		[59]	Western blot
	MHCC97 cells ++	[52]	Western blot; RT-PCR
		[59]	Western blot
	HuH-7	[59]	qPCR
	HepG2 cells	[53]	RT-PCR
	FIH and PHH (high expression but lower than *α*_1A_)	[53]	Immunofluorescence
	HepaRG cells (high expression but lower than *α*_1A_)		
	Mice hepatic stellate cells	[16]	RT-PCR
	Rat Kupffer Cells	[54]	Immunofluorescence
	Rat cholangiocytes	[60]	Immunohistochemistry
*β*_3_	LO2 cells	[52]	RT-PCR
	HepG2 cells	[52]	RT-PCR
	MHCC97 cells	[59]	RT-PCR
	Rat Kupffer Cells	[54]	Immunofluorescence
	Activated human hepatic stellate cells	[17]	RT-PCR; Western blot

ADR: adrenergic receptor; RT-PCR: real-time-polymerase chain reaction; RNA-seq: RNA sequencing; qPCR: quantitative polymerase chain reaction; PHH: primary human hepatocytes in culture.

## 5. Physiological and Metabolic Roles of Catecholamines in the Liver

### 5.1. Glucose Metabolism

**Figure 4:**
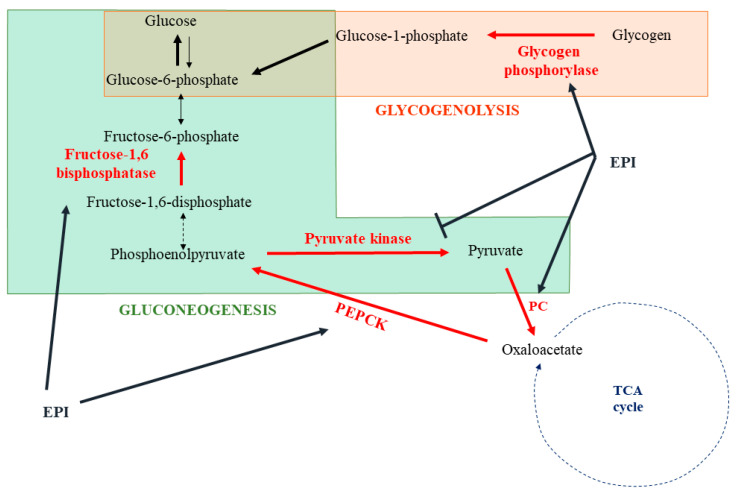
Impact of epinephrine on hepatic gluconeogenesis and glycogenolysis. Epinephrine (EPI) activates or inhibits some metabolic enzymes involved in glycogenolysis and gluconeogenesis (→ activation; —| inhibition). EPI: epinephrine; PC: pyruvate carboxylase; PEPCK: phosphoenolpyruvate carboxykinase; TCA cycle: tricarboxylic acid cycle.

One of the best-known actions of catecholamines is their capacity to induce glycogenolysis and gluconeogenesis in response to stress in order to generate energy substrates (Figure 4). The liver is the main organ implicated in this response. Depending on the species, *α*_1_-ADR, *β*_2_-ADR, or both receptors could be involved [61,62,63,64]. In human hepatocytes, catecholamines rapidly activate glycogenolysis by the stimulation of glycogen phosphorylase [65]. This was confirmed in rat hepatocytes and associated with an inactivation of the glycogen synthase [66]. In the second phase of catecholamine action, a shift to gluconeogenesis, with lactate and alanine as main substrates, is observed [67,68,69]. The origin of this shift remains not fully understood and may involve a glycogen depletion. At the molecular level, catecholamines induce post-translational modifications or direct modulation of the expression of several enzymes to stimulate gluconeogenesis. As such, epinephrine induces the phosphorylation and activation of fructose biphosphatase, which converts fructose 1,6-diphosphate into fructose-6-phosphate [70]. An increase in the pyruvate carboxylase activity, allowing the transformation of pyruvate in oxaloacetate, has also been described [71]. Interestingly, epinephrine enhances the expression of phosphoenolpyruvate carboxykinase (PEPCK), which facilitates the use of oxaloacetate to synthesize phosphoenolpyruvate [72,73]. Furthermore, phosphorylation of the pyruvate kinase by epinephrine-induced signaling leads to an inhibition of this enzyme, favoring the use of phosphoenolpyruvate to the synthesis of glucose [74].

Glucose metabolism is mainly observed in the periportal hepatocytes. However, depending on the nutritional status, a dynamic adaptation of the gene expression of enzymes involved in glucose metabolism is observed. For example, PEPCK is mainly expressed in periportal hepatocytes when rats are in a fed state [75]. During fasting, PEPCK is rapidly induced in periportal hepatocytes as well as in pericentral hepatocytes, with a diminution of the gradient expression compared to the fed state [75]. It could be interesting to evaluate whether a zonation is present for the expression of the ADR in order to understand these rapid adaptations to the nutritional status.

### 5.2. Fatty Acid Metabolism

In addition to their role in the production of glucose as an energy substrate in response to stressful situations, catecholamines facilitate the *β*-oxidation in hepatocytes in order to produce ATP and ketone bodies. First of all, epinephrine inhibits the secretion of triglycerides (TGs) from hepatocytes [76]. This effect was confirmed in an in vivo rat model of liver denervation as well as in a perfused rat liver model [77,78]. Appealingly, catecholamines might also favor the breakdown of hepatic intracellular TG into fatty acids. This effect has been described in the human Hep3B hepatoma cell line as well as in rat primary hepatocytes and involved a *β*_2_-ADR-cAMP-PKA-dependent mechanism [79]. Activation of this signaling pathway results in the phosphorylation and recruitment of hormone-sensitive lipase and adipose triglyceride lipase to lipid droplet surfaces, where they can liberate the free fatty acids from TG. Free fatty acids can then undergo *β*-oxidation. Interestingly, catecholamines have also been described to directly regulate long-chain fatty acid oxidation [80]. In rat primary hepatocytes, dopamine, by a *β*-ADR activation, increases the expression of carnitine palmitoyltransferases (CPTs), a crucial enzyme for the entrance of fatty acids into the mitochondria [81]. This induction is correlated with increased *β*-oxidation and ketogenesis. These results are in accordance with studies showing that by submitting rats to liver noradrenergic denervation, a decrease in the CPTI and II activities is observed [82]. This effect is not found in rats undergoing adrenodemedullectomy, strengthening a role for norepinephrine and sympathetic fibers in this regulation. Furthermore, catecholamines induce the phosphorylation of the acetyl-CoA carboxylase through an *α*-ADR mechanism [83]. This phosphorylation inhibits this enzyme, avoiding the transformation of acyl-CoA in malonyl-CoA. The accumulation of acyl-CoA in hepatocytes can then promote the *β*-oxidation of free fatty acid instead of their esterification into TG.

In summary, catecholamines could limit the secretion of VLDL (very low density lipoprotein)-TG in plasma and favor the degradation of TG in free fatty acids. These free fatty acids could then be used to produce energy through the *β*-oxidation pathway.

### 5.3. Catecholamines and Liver Regeneration

**Figure 5:**
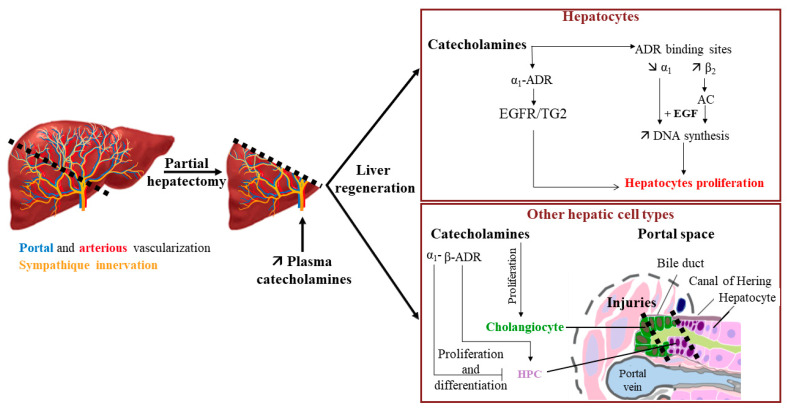
Impact of catecholamines on liver regeneration. Increase of plasma catecholamines after partial hepatectomy can participate in liver regeneration by enhancing the EGF effect on hepatocyte proliferation and through the proliferation/differentiation of various hepatic cell types (→ activation; —| inhibition). AC: adenylate cyclase; ADR: adrenergic receptor; EGF: epidermal growth factor; EGFR: EGF receptor; HPC: hepatic progenitor cells; TG2: transglutaminase 2. Adapted from [94].

The elevation of catecholamines in plasma is quickly observed after partial hepatectomy, suggesting that besides their effects on metabolism, they could also play a role in hepatocyte proliferation during liver regeneration (Figure 5) [84,85]. This hypothesis is strengthened by the fact that an *α*_1_-ADR stimulation could enhance the effect of EGF on the DNA synthesis in rat primary hepatocytes [86,87]. This latter effect is suspected to involve transglutaminase 2 (TG2), an enzyme owning both transamidation and GTPase activities. It has been described that the TG2 transamidation of the EGF receptor (EGFR) leads to a decrease in EGFR activation and thus to an inhibition of hepatocyte proliferation [88]. However, *α*_1_-ADR is able to interact with TG2, and its activation by norepinephrine shifts the transamidation activity of TG2 to GTPase activity, thereby preventing EGFR transamidation. This results in an upregulation of EGFR activity and perivenous hepatocyte proliferation [89].

An enhancement of the *β*_2_-ADR binding site density in rat hepatocytes is also observed upon partial hepatectomy compared to hepatocytes from sham control [56,90]. This increase begins 4 h after resection to reach a maximum 48 h after and is associated with a slight diminution of the *α*_1_-ADR binding site density. These results were in accordance with the higher increase of the AC activity induced by epinephrine in partial rat hepatectomies compared to sham rat liver homogenates [91]. Furthermore, isoproterenol, a *β*-adrenergic agonist, at low concentration (10^−10^ to 10^−8^ M) and in the presence of epidermal growth factor (EGF), dexamethasone, and insulin, increases DNA synthesis in rat primary hepatocytes isolated after partial hepatectomy. However, *β*-ADR activation has also been described to exert an inhibitory effect in the late G1 phase [92].

A sex difference has also been described concerning liver regeneration since catecholamines effects were mediated by both *α*- and *β*-ADRs in female rats, whereas only *α*-ADR seems implicated in male rats [93].

Interestingly, catecholamines could also act on other liver cells during liver regeneration. For example, catecholamines and adrenoceptor agonists favor the proliferation of cholangiocytes [55,60]. This suggests a beneficial action of catecholamines during the formation of lesions of bile ducts. Additionally, last but not least, catecholamines have been described to play a role in the conservation of the hepatic progenitor cell (HPC) pool. Indeed, in the presence of a severe injury, and when replication from differentiated hepatocytes is not sufficient to repair the liver damage, liver regeneration can also involve the proliferation and differentiation of HPC. Surprisingly, in a mouse model fed with an antioxidant-depleted diet, antagonism of *α*_1_-ADR by prazosin or chemical sympathectomy induced by 6-hydroxy-dopamine had a positive effect on liver injury by increasing the pool of HPC, suggesting that catecholamines could control it [95]. On the other hand, the use of the *β*-adrenoceptor agonist isoproterenol in acetaminophen-induced liver injuries in a dopamine *β*-hydroxylase-deficient mouse model allowed the recovery of the HPC pool [96]. These results suggest an opposite action of *β*-ADR activation compared to *α*_1_-ADR activation on HPC expansion.

Taking together, these results suggest that catecholamines could have a positive role in liver repair when the regeneration process involves differentiated hepatocytes. It would be interesting to evaluate if variations in the expression of ADR are observed in HPC when major injuries occur in order to explain how catecholamines could favor the proliferation of the HPC pool in such situations.

## 6. Involvement of Catecholamines in Liver Diseases

### 6.1. Catecholamines and Drug-Metabolizing Enzymes Modulation

In addition to the role of the central noradrenergic system in the regulation of hepatic cytochrome P450 (CYP) expression by modulating the hypothalamic–pituitary–adrenal axis, few studies have described a direct impact of epinephrine on the expression of hepatocyte drug-metabolizing enzymes. For example, a 24 h treatment with epinephrine of primary rat hepatocytes in culture led to the cAMP-mediated repression of CYP2C11 mRNA [97]. We also observed the repression of CYP3A4 by treating primary human hepatocytes and the HepaRG hepatoma cell line with epinephrine [98]. This CYP3A4 repression was also observed on cryopreserved human hepatocytes treated every 24 h for 48 h by epinephrine and norepinephrine [99]. On the other hand, Daskalopoulos et al. described the induction of CYP3A1/2 by epinephrine in primary rat hepatocytes [100]. Additionally, the repression of several drug transporters by a *β*_2_-ADR/cAMP mechanism after the treatment of primary human hepatocytes or the HepaRG cell line with epinephrine has also been described [53]. In this study, several sinusoidal influx transporters, such as NTCP and efflux transporters (MRP2, BSEP), are repressed, whereas MDR1 is found to be induced by epinephrine. However, to our knowledge, such phenomena have not been described in vivo. Hence, further investigations are warranted to determine whether these modulations are observed in pathophysiological situations in which catecholamines are highly secreted, such as pheochromocytoma, a tumor leading to an overproduction of catecholamines.

### 6.2. Catecholamines and Liver Dysfunction in Sepsis

Sympathetic system activation is observed during sepsis. Thus, in a rat model of sepsis, induced by cecal ligation and puncture, an increase in epinephrine, norepinephrine, and dopamine plasma concentrations was observed 5 h after the onset of sepsis [101,102]. The main source of norepinephrine in the early phase of sepsis in this model is the mesenteric tract [103]. These results are in accordance with the induction of tyrosine hydroxylase expression in the gut sympathetic nerve fibers observed during sepsis [104]. Several studies have shown that this early release of catecholamines in the portal vein is implicated in the initial hepatocellular dysfunction, notably by potentiating the production and secretion of proinflammatory cytokines (TNF*α*, IL-1*β*, IL-6) by Kupffer cells [103,104,105,106]. Activation of *α*_2_-ADR by norepinephrine is thus implicated in the secretion of TNF*α* by Kupffer cells [107,108,109], and the use of a specific *α*_2A_ antagonist reduced the inflammatory response and liver transaminases release in a rat model of sepsis following cecal ligation and puncture [110]. Thus, a high concentration of catecholamines may induce liver injury. Although little is known on the topic, at least one study has already described hepatic cytolysis with elevated transaminases and C-reactive protein (CRP) in a patient suffering from pheochromocytoma [111].

### 6.3. Catecholamines and Fatty Liver Diseases

The incidence of metabolic-associated fatty liver disease (MAFLD) is constantly rising in the Western world. Consumption of a highly caloric diet and/or limited physical activities are the main causes of these pathologies. The prevalence of these diseases is also known to be enhanced with aging. As previously mentioned, aging is characterized by an increase in *β*-ADR expression in rodents. This increase is correlated with an enhancement of AC activity in the presence of *β*_2_-agonists in liver homogenates from 24-month-old rats compared to younger ones [112]. As steatosis development is also linked to aging, the question arises about the role of the *β*-ADR in lipid accumulation in hepatocytes. In vivo experiments in old and young rats seem to confirm this hypothesis [112,113]. Furthermore, treatment with the long *β*_2_-agonist formeterol induces an increase in lipid hepatic content in mice [114]. Mechanisms allowing this steatosis involve the increased expression of enzymes implicated in TG synthesis, such as DGAT1 (diacylglycerol O-acyltransferase 1) and lipin-1, and of genes involved in lipid droplets formation (Cidea, Cidec, Plin2, -3, and -5, and Hilpa), concomitant to a reduction in VLDL-TG secretion.

In the same way, an increase in sympathetic activity has been described in patients with metabolic syndrome [115] and in a high-fat diet-induced obesity (HFD) rodent model [116]. This increased activity has been linked to higher liver TG and lipid droplet contents and correlated to an increased expression of genes involved in fatty acid uptake (CD36) and de novo lipogenesis (DGAT1 and DGAT2) [116]. However, in these studies, the measurement of the nerve activity has been done outside the hepatic lobe, while, in a more recent paper using the iDISCO technique, it has been described that the HFD regime leads to the reversible destruction of sympathetic fibers within the liver [1]. These disturbances in the sympathetic nerve fibers in mouse steatotic livers, as well as the degeneration in human steatohepatitis, have also been observed by Adori et al. [2]. In this last paper, they suggest that chronic sympathetic hyperexcitation is a key factor in this degeneration. Taken together, these experiments indicate a contribution of catecholamines in steatosis establishment.

### 6.4. Catecholamines and Fibrosis

The sympathetic system and norepinephrine play a role in liver fibrosis by acting on stellate cells [14]. Indeed, liver fibrosis induced by carbon tetrachloride (CCl_4_) is lower in rats after the sympathetic denervation or injection of the *α*_1_-ADR antagonist compared to the control group [117]. This phenomenon could be due to a decreased expression of collagen type I and of the tissue inhibitor of metalloproteinase 1 (TIMP1) in the livers of rats with denervation. In addition, carvedilol, an *α*- and *β*-non-selective antagonist, as well as doxazosin, an *α*_1_-ADR-antagonist, have been described to attenuate liver fibrosis induced by CCl_4_ in mice [118,119]. This hypothesis was partly confirmed in a leptin-deficient ob/ob mouse model that exhibits low plasma norepinephrine concentration. In these mice, the injection of norepinephrine leads to an increase in TGF-*β* and collagen type I-*α*_1_ as well as to liver fibrosis [120]. In addition, higher expression of *α*_1A_-ADR is observed in human cirrhotic liver compared to normal liver, and norepinephrine, through the activation of this receptor, increases the production of several proinflammatory chemokines, such as RANTES and IL-8, in human stellate cells [50]. Interestingly and in line with the recent observations in the hepatosteatosis described above [2], an analysis of biopsies from human normal or fibrotic livers following HBV/HCV infection or NASH revealed a correlation between the degree of fibrosis and the decrease of intrahepatic sympathetic nerve fibers [121]. Thus, the activation of sympathetic fibers could be involved in the initiation of fibrosis, whereas in advanced fibrosis, when intralobular denervation is observed, the implication of catecholamine production by stellate cells could be suspected.

Catecholamines also play a role in the development of alcoholic liver disease. Elevation of norepinephrine plasma concentrations is observed after alcohol consumption [122,123]. This enhancement is due to an increase in sympathetic activity as well as in the release of catecholamines by the adrenal medulla. Indeed, chronic exposure to ethanol leads to an induction of the main enzymes involved in catecholamine synthesis (TH, BDH, and PNMT) [124]. Thus, norepinephrine and epinephrine participate in the establishment of an inflammatory environment in the liver during ethanol exposure by activating the Kupffer cells as well as the stellate cells [16,109]. These mechanisms are thus implicated in the apparition of fibrosis and steatohepatitis during chronic exposure to alcohol [125].

### 6.5. Catecholamines and Liver Cancers

A link between stress or psychosocial factors and the incidence or increasing aggressiveness of cancer has been established for several types of cancer. Among the molecules that could be implicated in this process, catecholamines have been suggested since their production is increased in these situations [126,127]. In agreement with this hypothesis, catecholamines have been described to modulate tumor cell migration, angiogenesis, and the epithelial-to-mesenchymal transition (EMT) in various types of cancer, especially in breast and prostate cancers [128,129]. Since the beginning of the 1990’s, studies have pointed out that it may also be the case for liver cancers [130]. To our knowledge, Bevilacqua et al. were the first to introduce this concept by describing a difference in the density of adrenoceptors in the tumor tissue of human hepatocellular carcinoma (HCC) compared to the adjacent non-tumoral tissue [131]. This modulation was characterized by an increase in *β*_2_-ADR and a decrease in the *α*_1_-ADR expression in tumor tissue. These results were confirmed by Kassahum et al. [57,132], and later, the downregulation of the *α*_1A_-ADR in HCC was assigned to aberrant hypermethylation in its promoter [51]. More recently, the implication of the microRNA miR-3682, overexpressed in HCC tissues and targeting the *α*_1_-ADR, has been incriminated in this repression [133]. In addition, the high density of sympathetic nerve fibers in HCC is also correlated with poor prognostic results [54].

Several mechanisms explaining the role of catecholamines in HCC progression have been proposed:

I. Epinephrine activation of the *β*_2_-ADR expressed by tumor hepatocytes initiates the Akt signaling pathway and leads to autophagy inhibition, which consequently stabilizes the transcription factor hypoxia inducible factor 1 subunit *α* (HIF-1*α*). This stabilization boosts glucose metabolism, notably by increasing hexokinase-2 (HK2) expression, and favors cell growth and tumor progression [58]. In addition, Akt signaling pathway activation by *β*_2_-ADR has been involved in the nuclear translocation of the oncoprotein YB-1(Y binding protein 1), leading to EMT and *β*-catenin induction [134];
II. Both *α*_1A_- and *β*_2_-ADR activations are responsible for an increase in cell invasion through EGFR transactivation [135];
III. *β*_2_-ADR mediates anoikis inhibition in several hepatic cancer cell lines [135];
IV. By acting on the *β*-ADR of the hepatic tumor cells, norepinephrine induces the secretion of chemokines such as CXCL2. In turn, CXCL2 is able to recruit splenic myeloid cells in the tumor tissue and favor hepatic tumor progression [136];
V. Catecholamines can also act on the cells of the tumor microenvironment. Norepinephrine, by binding the *α*_1_-ADR of Kupffer cells, increases the secretion of IL-6 and TGF-*β*. This inflammatory environment promotes tumor development [54]. Hepatic stellate cells stimulated by norepinephrine favor HCC progression by enhancing the proliferation, EMT, and stemness properties of HCC cells, notably through the secretion of secreted frizzled-related protein 1 (sFRP1), which activates the Wnt16B/*β*-catenin pathway in hepatic cancer cells [137].

Interestingly, the repression of MAOA, an enzyme involved in the degradation of norepinephrine and epinephrine, is observed in the tumor tissue and is associated with the invasiveness and poor prognostic of several cancers, including HCC and cholangiocarcinoma [135,138,139,140]. This repression could be due to an epigenetic regulation since the hypermethylation of the promoter of this gene is observed in tumors. Inflammation may also be implicated since a specificity protein 1 (SP1) binding site is present in the MAOA promoter. Indeed, the transcription factor SP1 induces MAOA expression, but this is abolished by IL-6, which prevents SP1 binding to its consensus sequences by enabling the R1 repressor to bind instead [135,140]. Interestingly, hypoxia, which is observed in tumors, is also known to repress the expression of this enzyme, as observed in the hepatoma cell line HepaRG [141]. This repression may lead to an increase in norepinephrine concentration within the tumor tissue, thus enhancing the local effects of this catecholamine.

Considering these studies, the higher expression of *β*_2_-ADR in HCC has been suggested as a prognostic biomarker for lower survival rates as well as for tumor recurrence [59]. Furthermore, HK2 and ADRB2 overexpression has been found to positively correlate and associate with poor prognosis in HCC patients [142]. Serum catecholamine concentrations associated with the HAMA score, an evaluation of anxiety, have also been identified as potential prognostic markers in HCC patients [143]. With regard to these results, the use of beta-blocker in cancer has been suggested as adjuvant therapy, and several publications have highlighted a beneficial effect of these drugs, notably on melanoma and breast cancers [144,145]. As *β*-blockers are currently used in cirrhosis in order to reduce portal hypertension and the risk of hemorrhage in esophagus varices, investigating their role in HCC could be relevant. To our knowledge, only five retrospectives studies have evaluated their effects and have highlighted a positive impact of propranolol, a non-selective *β*-blocker, on the occurrence of HCC in patients with hepatitis C virus (HCV)-associated cirrhosis [146], alcoholic cirrhosis [147], or with no distinction of HCC etiology in patients with cirrhosis [148]. The use of propranolol prescription before HCC diagnosis improved the survival rate in a Swedish cohort of 2104 HCC patients [149]. Finally, the use of three distinct non-selective *β*-blockers (carvedilol, nadolol, and propranolol) was associated with a lower risk of HCC incidence in a large cohort of 107.428 HCC American patients with cirrhosis [150]. Hence, *β*-blockers appear as a promising pharmacological strategy in lowering HCC incidences; whether other *β*-ADR or *α*-ADR antagonists would exert beneficial effects remains to be determined.

## 7. Therapeutic Implications

Adrenergic antagonists are well-known drugs for which we have certain hindsight in their use in medicine. The majority of these molecules are non-selective *β*-blockers, such as propranolol or nadolol. Selective *α*_1_-ADR antagonists, such as prazosin or doxazosin, are also used clinically to treat hypertension, prostatic hyperplasia, or post-traumatic stress disorders. Carvedilol is a *β*-ADR and *α*_1_-ADR receptor antagonist used in the treatment of heart failure. As discussed in paragraph 6.5, several retrospective studies have been carried out on these molecules in the context of HCC. Furthermore, studies performed on animal models with fibrosis or cirrhosis demonstrated that carvedilol could act on tumor processes such as inflammation, matrix remodeling, resistance to cell death, or even angiogenesis [151]. Carvedilol appears to be hepatoprotective against fibrosis by limiting the effects of hepatic stellate cells and decreasing types I, III, and IV collagen deposits and the production of TGF-*β*1 [118,152]. Retrospective studies based on clinical data registries in order to follow patients under these therapies for other pathologies, such as high blood pressure, for example, could give an idea of their benefits in the apparition of liver diseases.

## 8. Conclusions

**Figure 6:**
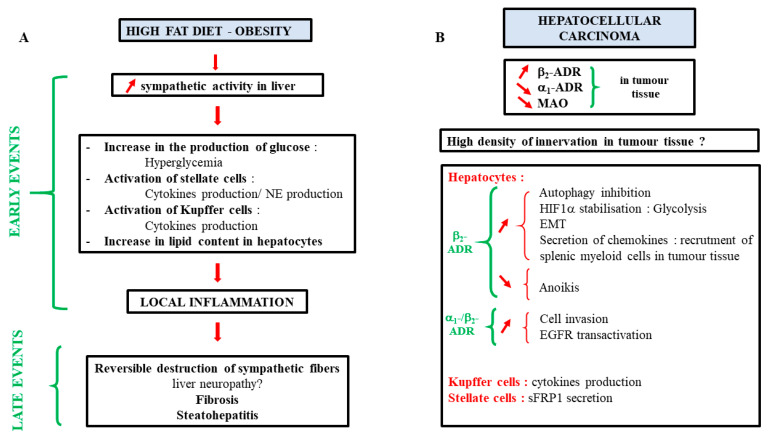
Summary of catecholamines liver effects during a high fat diet or obesity (**A**) and on HCC progression (**B**). (**A**) During high fat diet or obesity, an increase in liver sympathetic activity is observed during the onset of the pathology. This increase could lead to early events characterized by an increase in gluconeogenesis, an activation of stellate cells, cytokine production by Kupffer cells, and an increase in lipid content in hepatocytes. All of these phenomena could induce a local inflammation responsible for the reverse destruction of liver sympathetic fibers as well as fibrosis and steatohepatitis. These results suggest that *α*/*β*-antagonists could have a beneficial effect in the early stages of steatosis/fibrosis. (**B**) In hepatocellular carcinoma, a decrease in the expression of *α*_1_-ADR and MAO and an increase in the expression of *β*_2_-ADR are observed. High sympathetic innervation of the tumor tissue has also been observed but remains to be validated by other studies. In vitro and in vivo studies have also shown that several processes involved in cancer progression are activated by catecholamines. MAO: monoamine oxidase, EMT: epithelial to mesenchymal transition, sFRP1: secreted frizzled-related protein 1.

As described in this review, catecholamines play important roles in many aspects of liver physiology. However, interrogations are still present, notably on their implication in several pathophysiological processes such as the development of fibrosis, MAFLD, or cancer progression. A summary of the current knowledge is presented in Figure 6. Increasing works about their involvement in these diseases may lead to new therapies or adjuvant treatments. As antagonists of ADR are well-known drugs introduced on the market for a long time now, their use in these pathologies could be facilitated.

## Figures and Tables

**Table 1:** Expression of the dopamine receptor mRNAs in human hepatocytes in primary human hepatocytes in culture (PHH) and in four hepatoma cell lines (HepG2, HepaRG, HuH-7, HBG-BC2).

	PHH	HepG2	HepaRG Progenitors	HepaRG Hepatocytes	HepaRG Cholangiocytes	HuH-7	HBG-BC2
*DRD1*	35.1 ± 2.00	34.1 ± 1.40	35.5 ± 1.43	34.0 ± 1.42	36.6 ± 1.74	35.8 ± 2.06	31.7 ± 0.85
*DRD2*	37.1 ± 1.72	35.5 ± 1.89	34.1 ± 1.85	36.3 ± 0.71	35.5 ± 1.31	35.4 ± 1.54	36.6 ± 1.57
*DRD3*	37.0 ± 1.38	37.9 ± 0.89	37.1 ± 1.52	37.4 ± 0.91	37.8 ± 1.31	35.8 ± 2.02	37.3 ± 0.59
*DRD4*	30.8 ± 1.19	28.3 ± 0.52	29.5 ± 0.43	29.3 ± 0.42	29.9 ± 0.74	30.5 ± 0.27	29.5 ± 0.75
*DRD5*	30.8 ± 0.82	28.1 ± 0.70	29.2 ± 0.32	29.1 ± 0.29	30.4 ± 0.92	30.5 ± 0.70	31.2 ± 0.92

Expression of dopamine receptor (DRD) mRNAs has been analyzed by real-time quantitative polymerase chain reaction (5 ng of cDNA) in progenitors HepaRG cells (4 days of culture), differentiated HepaRG-hepatocytes, HepaRG-cholangiocytes, as well as in HepG2, HuH-7, HBG-BC2 cells and in PHH. mRNAs’ expression is represented by the cycle threshold (CT). CT values above 30 (in grey) are considered as not expressed in the cells. All values are the mean of 3 independent experiments in triplicates, except for cholangiocytes-HepaRG (3 experiments with biological duplicates) and PHH (5 experiments with biological triplicates).

## Supplementary Materials

The following supporting information can be downloaded at: https://www.mdpi.com/article/10.3390/cells11061021/s1, Figure S1: Expression of the dopamine receptors mRNAs in primary human hepatocytes in culture (PHH) and in four hepatoma cell lines (HepG2, HepaRG, HuH-7, HBG-BC2); Table S1: Primer sequences used for qPCR.
