# Supplementary material for: The Role of Catecholamines in Pathophysiological Liver Processes
Source: Cells. 2022 Mar 17;11(6):1021. doi: 10.3390/cells11061021 (PMC8947265; doi:10.3390/cells11061021)
Supplement: Supplementary file 1 [file cells-11-01021-s001.zip › cells-1603421-supplementary.pdf]

Supplementary Materials:

Supplementary Table S1. Primer sequences used for qPCR.

	Primer sequences (F: Forward / R: Reverse)
DRD1	F: AGGGACTTCTCTGTTCGTATCC<br>R: GGAACCTGATAACGGCAGCA
DRD2	F: CTCTTCGGACTCAATAACGCAG<br>R: GACGATGGAGGAGTAGACCAC
DRD3	F: AGAAGGCAACCCAAATGGTGG<br>R: TGTCGTGGCACTGTAAAGCTC
DRD4	F: CCACCAACTCCTTCATCGTG<br>R: TCGGAGTAGACGAAGAGCG
DRD5	F: CTCAACTGGCACAGGGAC<br>R: GATGAGCGAGGAAGAGATG
GAPDH	F: TGCACCACCAACTGCTTAGC<br>R: GGCATGGACTGTGGTCATGAG

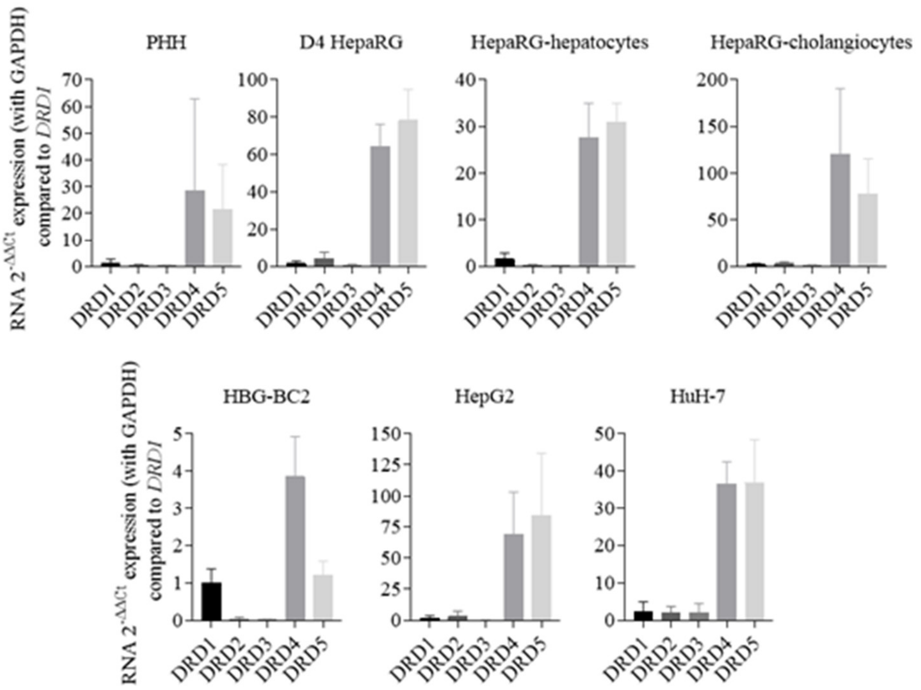

Supplementary Figure S1. Expression of the dopamine receptors mRNAs in primary human hepatocytes in culture (PHH) and in four hepatoma cell lines (HepG2, HepaRG, HuH-7, HBG-BC2). Expression of dopamine receptors (DRD) mRNAs has been analyzed by real-time quantitative polymerase chain reaction (5 ng of cDNA) in progenitors HepaRG cells (4 days of culture), differentiated HepaRG-hepatocytes, HepaRG-cholangiocytes, as well as in HepG2, HuH-7, HBG-BC2 cells, and PHH. mRNAs expressions were measured by the $2^{-\Delta\Delta CT}$ method using GAPDH as the housekeeping gene, and expression values were compared to DRD1 expression. All values are the mean of 3 independent experiments in triplicates, except for cholangiocytes-HepaRG (3 experiments with biological duplicates) and PHH (5 experiments with biological triplicates).
